# Solitary Condyloma Acuminatum of the Dorsal Tongue: An Atypical Presentation and Diagnostic Challenge

**DOI:** 10.7759/cureus.86510

**Published:** 2025-06-21

**Authors:** Francesco Valente, Pierluigi Valente, Andrea Sbrenna, Lapo Sbrenna, Vittorio Unfer

**Affiliations:** 1 Department of Oral Implantology, San Damiano Dental Clinic, Rome, ITA; 2 School of Dentistry, Vita-Salute San Raffaele University, Milano, ITA; 3 Department of Dentistry, Humanis Dental Center, Perugia, ITA; 4 School of Dentistry, Alma Mater Studiorum - Bologna University, Bologna, ITA; 5 Department of Obstetrics and Gynecology, Unicamillus International Medical University, Rome, ITA

**Keywords:** condyloma acuminatum, dorsal tongue, human papilloma virus dna, oral hpv lesion, solitary papillary lesion

## Abstract

Solitary papillary lesions of the oral cavity may present diagnostic challenges, particularly when arising in uncommon locations, such as the dorsal surface of the tongue, and in the absence of other mucocutaneous involvement. We report the case of a 55-year-old male patient with a small, asymptomatic, sessile lesion on the dorsum of the tongue, measuring approximately 2 mm in height. The lesion appeared white, standing in sharp contrast to the surrounding erythematous mucosa, drawing attention despite its diminutive size. Clinical appearance suggested a benign proliferation, and human papillomavirus (HPV) DNA testing via real-time polymerase chain reaction (PCR) for a broad panel of high- and low-risk genotypes returned negative results. However, histopathological examination revealed features consistent with condyloma acuminatum, including papillomatosis, acanthosis, and scattered koilocytosis in the spinous layer. This discordance between molecular and histological findings underscores the diagnostic limitations of HPV testing and the complexity of interpreting viral clearance, which may reflect true elimination, low viral load below the assay’s detection threshold, latency, or inadequate sampling. These considerations highlight the importance of integrating clinical, histological, and molecular findings in the evaluation of atypical oral lesions.

## Introduction

Papillary lesions of the oral cavity represent a heterogeneous spectrum of benign proliferations, often posing diagnostic challenges due to overlapping clinical and histopathological features [1]. Human papillomavirus (HPV) infection is extremely common in the general population. According to the Italian National Institute of Health (Istituto Superiore di Sanità), up to 80% of sexually active women may contract an HPV infection during their lifetime, with over 50% becoming infected with a high-risk oncogenic type. Similarly, epidemiological studies report that approximately one-third of men are infected with at least one HPV genotype, and nearly one-fifth with a high-risk oncogenic type. While most HPV-related research has historically focused on cervical and anogenital infections, the oral cavity is increasingly recognized as a relevant site of HPV-associated lesions. HPV-related lesions, including squamous papilloma, condyloma acuminatum, and verruca vulgaris, are among the most frequently identified causes of benign exophytic growths in the oral cavity. These lesions typically affect mucosal sites such as the cervix, anus, and oropharynx, with the exception of verruca vulgaris, which is predominantly cutaneotropic. However, non-viral reactive epithelial hyperplasias, such as the lingual mucosal horn, may mimic their appearance, particularly when occurring in atypical sites like the dorsal tongue [2]. HPV-related lesions are more frequently observed in non-keratinized oral mucosal sites, including the labial mucosa, floor of the mouth, soft palate, and lingual frenum, where the epithelial barrier is thinner and local immune defenses are comparatively reduced [3]. In such cases, the clinical presentation alone may be insufficient for accurate classification, necessitating histological and molecular correlation. While oral condyloma acuminatum is a benign lesion with low oncogenic potential, failure to recognize and treat it may lead to persistence, growth, or risk of viral transmission. Herein, we describe a case of a small, solitary, asymptomatic papillary lesion on the dorsal tongue of an immunocompetent adult. Despite negative HPV DNA testing by real-time polymerase chain reaction (PCR), histopathological examination revealed features consistent with condyloma acuminatum. This case underscores the importance of comprehensive diagnostic assessment and highlights the potential for discordance between molecular and microscopic findings in oral epithelial lesions.

## Case presentation

**Figure 1:**
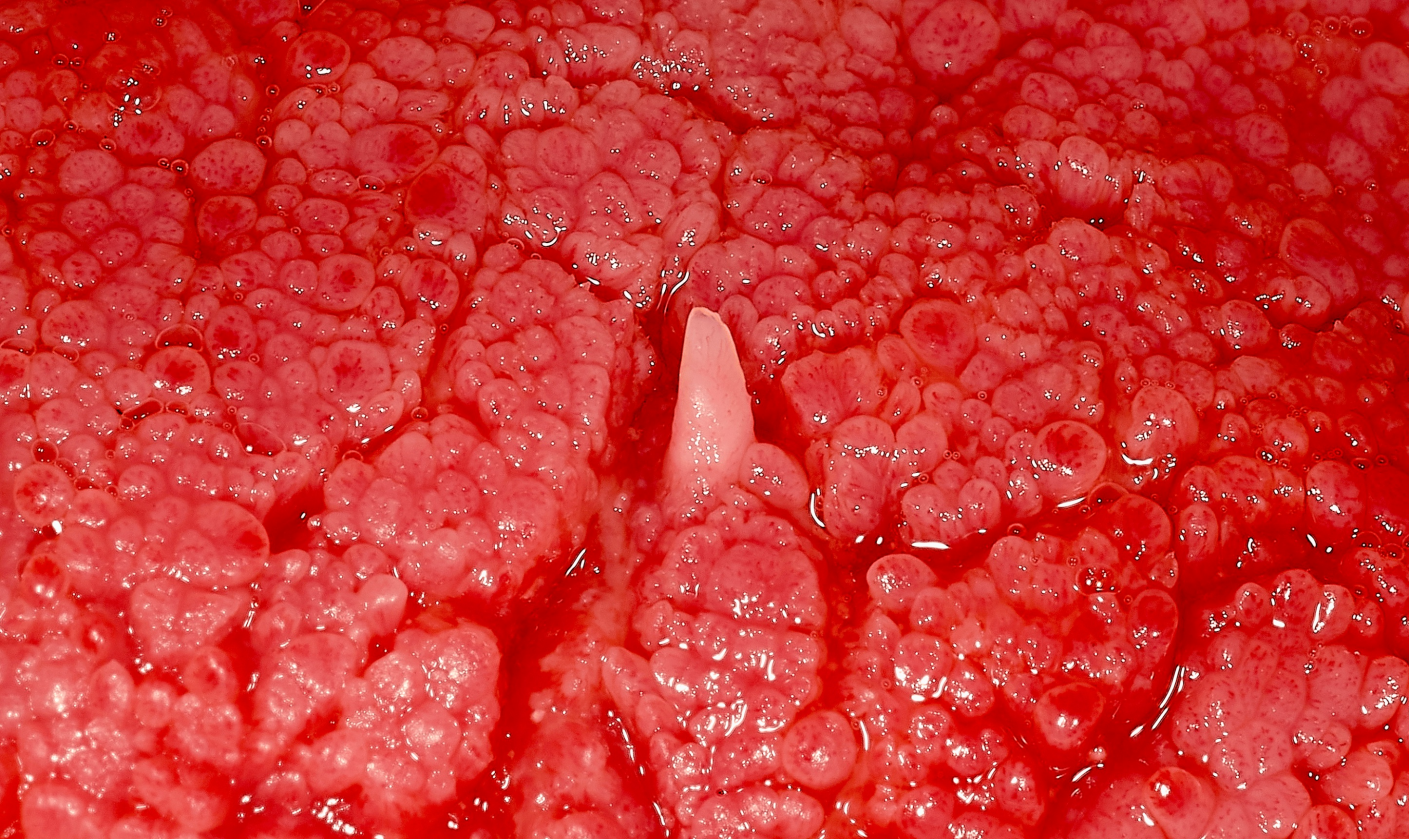
Solitary whitish papillary lesion with smooth surface on the dorsal tongue.

A 55-year-old male patient presented for a routine dental examination at our clinic, San Damiano Dental Center in Rome, Italy, in March 2025. He reported no history of systemic illness, immunosuppression, or prior HPV-associated lesions. He also denied high-risk sexual behaviors, genital warts, or cutaneous lesions on the hands or other body sites. Intraoral examination revealed a solitary, sessile, well-demarcated papillary lesion situated on the mid-dorsal surface of the tongue, measuring approximately 2 mm in height and less than 1 mm in diameter (Figure 1).

**Figure 2:**
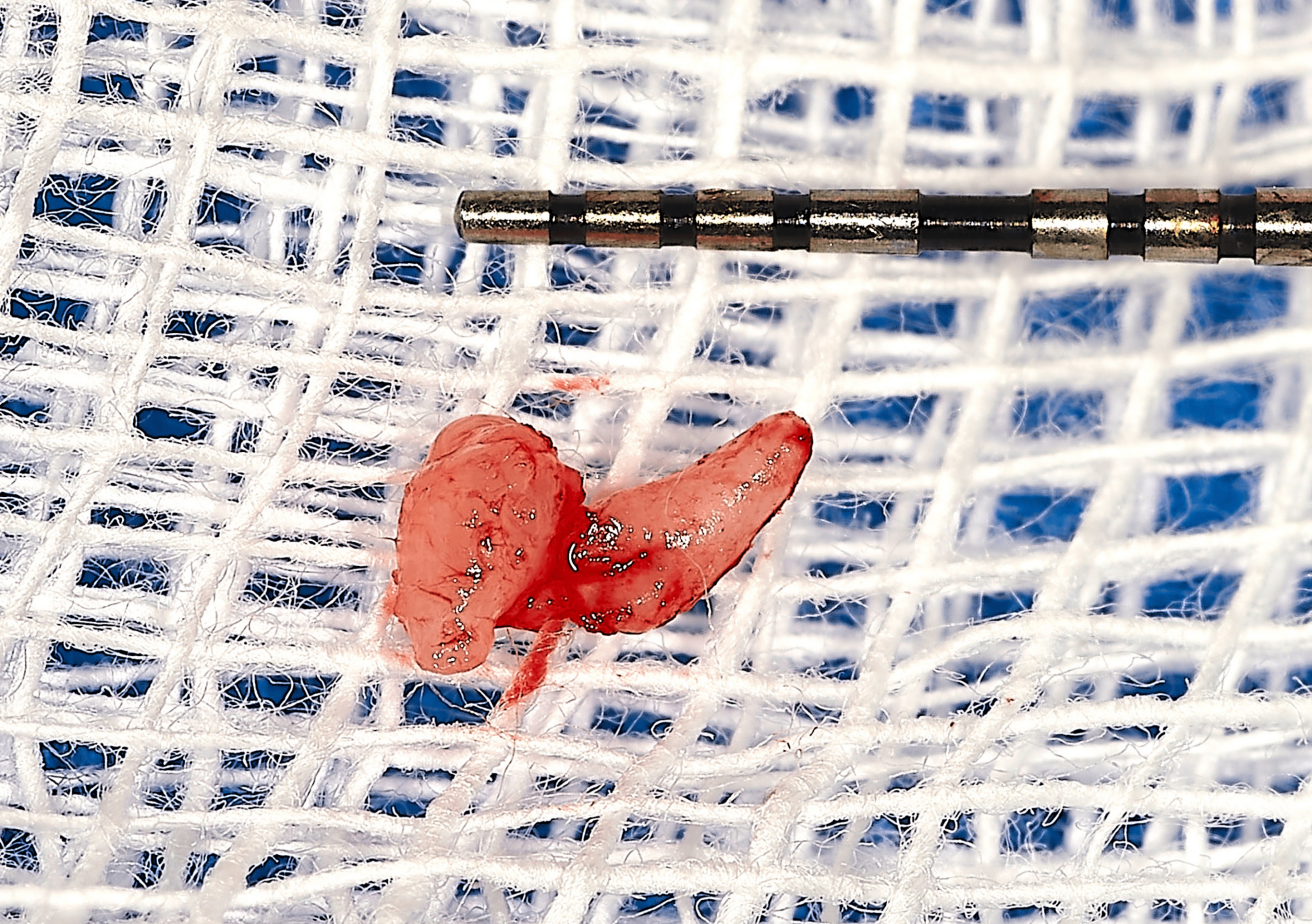
Excised lesion approximately 2 mm in height; periodontal probe for scale.

The lesion was firm on palpation, asymptomatic, and exhibited a smooth, whitish surface that contrasted sharply with the surrounding reddish mucosa. The patient was completely unaware of the lesion prior to its identification during the examination. A clinical differential diagnosis included oral squamous papilloma, verruca vulgaris, condyloma acuminatum, and a non-viral reactive mucosal horn. The lesion was excised under local anesthesia and submitted for histopathological evaluation (Figure 2).

The excised lesion was fixed in formalin, embedded in paraffin, and sectioned for microscopic examination. Hematoxylin and eosin (H&E) staining was used to evaluate tissue architecture and cellular morphology. Immunohistochemical (IHC) staining (e.g., Ki-67, p16) was not performed in this case. A dry swab was collected from the lesion surface prior to surgical excision and submitted for HPV DNA analysis. Testing was performed using Real-Time PCR targeting the L1 region of the HPV genome (Altamedica Artemisia, Rome, Italy), with genotyping for a broad panel of both high-risk (HPV 16, 18, 26, 31, 33, 35, 39, 45, 51, 52, 53, 56, 58, 59, 66, 68, 69, 70, 73) and low-risk (HPV 6, 11, 32, 34, 40, 42, 43, 44, 54, 61, 72, 81) types. HPV types 2 and 4 were not included in the laboratory’s test panel; these subtypes are commonly associated with verruca vulgaris, and their exclusion limits the ability to fully rule out this differential diagnosis based solely on molecular data. The result was negative for all genotypes tested.

**Figure 3:**
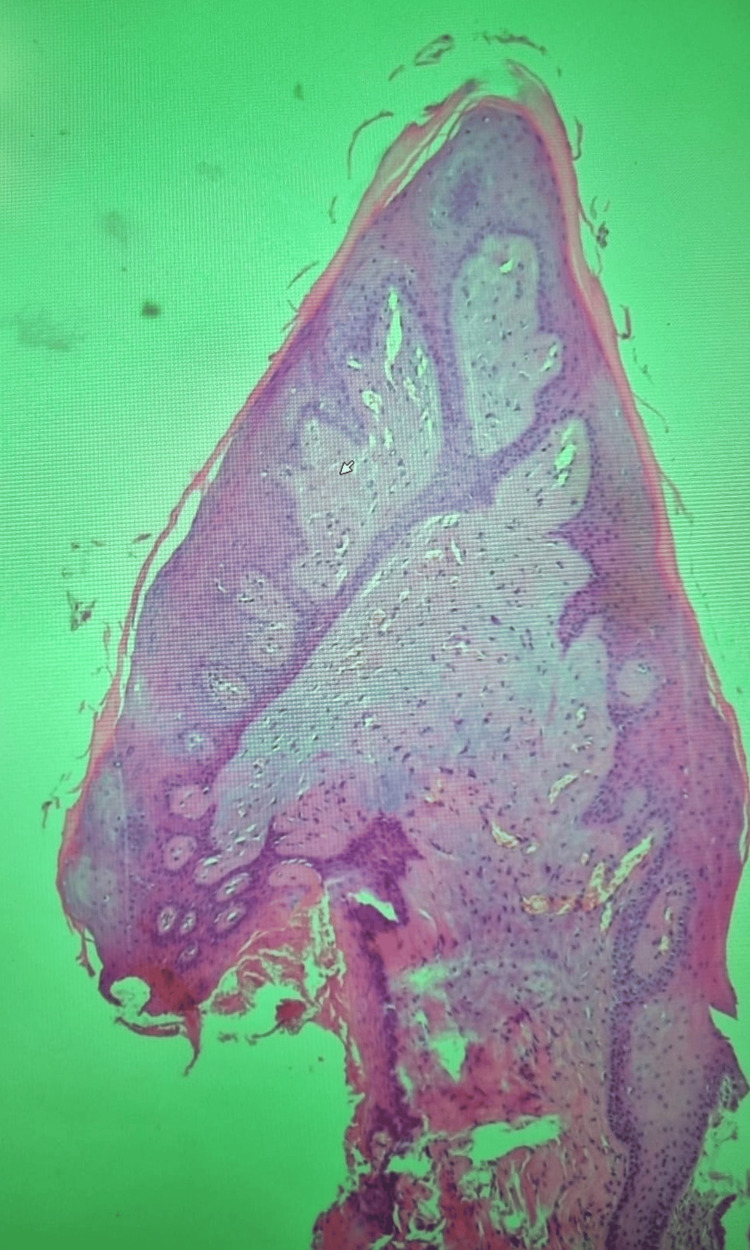
Low-power histological view of the excised lesion (hematoxylin & eosin stain, original magnification ×40). The epithelium is stratified squamous and shows acanthosis with broad papillary projections consistent with papillomatosis.

Histological examination revealed a well-circumscribed, exophytic epithelial proliferation characterized by acanthosis and papillomatosis (Figure 3).

**Figure 4:**
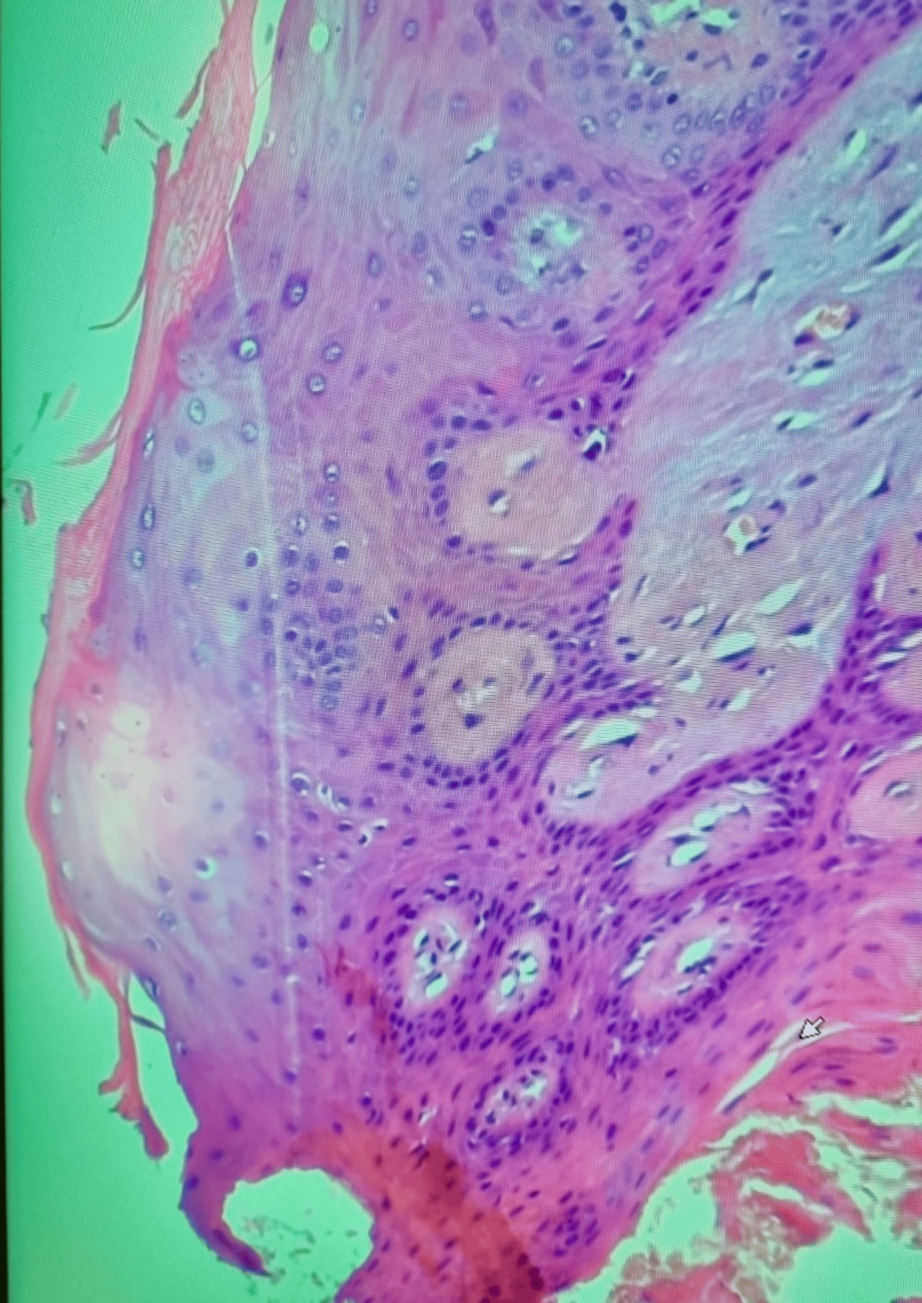
Intermediate-power histological view of the excised lesion (hematoxylin & eosin stain, original magnification ×100). The stratified squamous epithelium shows acanthosis and mild papillomatosis. Numerous keratinocytes in the upper spinous layer display classic features of koilocytosis, including enlarged, irregular, hyperchromatic nuclei with clear perinuclear halos.

The superficial epithelial layers displayed scattered koilocytotic changes, with perinuclear clearing and nuclear irregularities, particularly within the spinous layer. No evidence of epithelial dysplasia or parakeratosis was observed. Despite these histopathological features being consistent with condyloma acuminatum, real-time PCR analysis for a broad panel of high- and low-risk HPV genotypes, including types 6, 11, and 40, yielded a negative result. Histological examination demonstrated stratified squamous epithelium with acanthosis and papillomatosis, along with broad, blunt rete ridges. A thick orthokeratotic stratum corneum was observed, with an intact granular layer and no parakeratosis. Scattered koilocytes (keratinocytes with perinuclear clearing and nuclear irregularities) were identified within the spinous layer (Figure 4).

**Figure 5:**
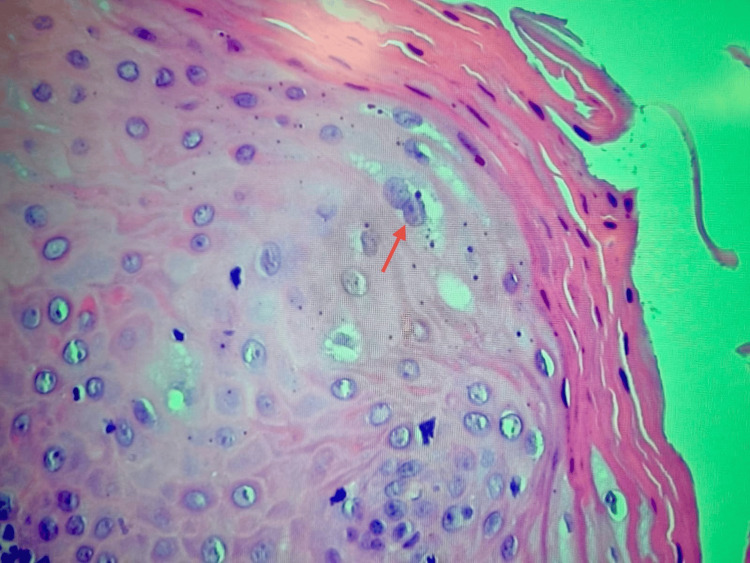
High-power histological view of the spinous layer (hematoxylin & eosin stain, original magnification ×200). The stratified squamous epithelium exhibits scattered koilocytes, characterized by prominent perinuclear halos and nuclear irregularities, suggestive of human papillomavirus (HPV)-induced cytopathic effect. These changes are mainly located in the upper spinous layers. Several koilocytes show binucleation (red arrow), nuclear size variation, and irregular chromatin distribution, all cytologic alterations strongly suggestive of HPV infection.

Mitotic activity was confined to the basal layer, and no epithelial dysplasia was noted. The squamous epithelium demonstrates orderly stratification and preserved maturation, with mitotic figures limited to the basal layer. Notably, several keratinocytes within the spinous layer exhibit perinuclear clearing and nuclear irregularities, consistent with koilocytosis. Several keratinocytes also exhibit binucleation, a cytopathic alteration frequently observed in HPV-associated lesions (Figure 5).

**Figure 6:**
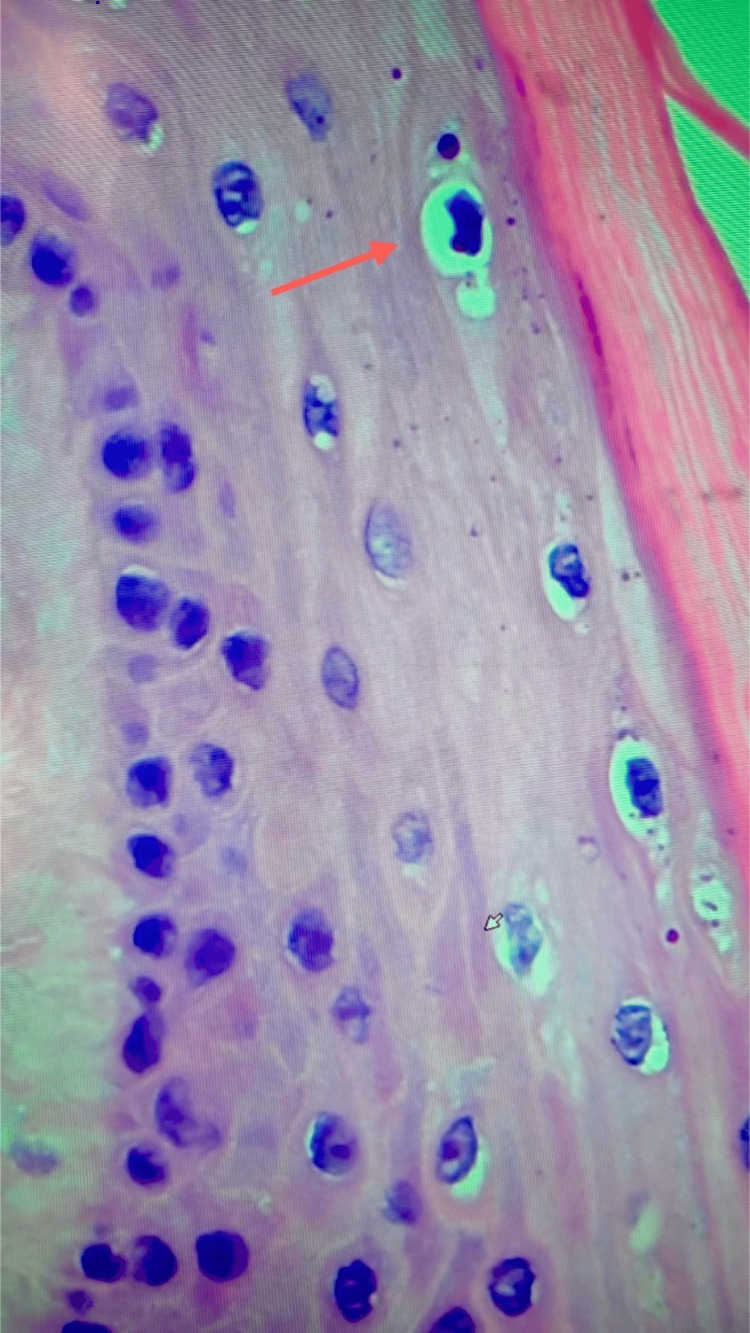
High-power histological view of the spinous layer (hematoxylin & eosin stain, original magnification ×200). The stratified squamous epithelium exhibits scattered koilocytes (red arrow), characterized by prominent perinuclear halos and nuclear irregularities, suggestive of human papillomavirus (HPV)-induced cytopathic effect.

The combined presence of koilocytosis, nuclear size variation, and binucleation provides robust histopathological evidence of HPV infection. In the absence of molecular confirmation, the constellation of koilocytosis and epithelial papillomatosis, alongside the absence of pronounced hypergranulosis and hyperkeratosis, favors a diagnosis of condyloma acuminatum. This highlights a potential diagnostic discordance and underscores the limitations of HPV DNA detection, particularly in small or histologically regressed lesions (Figure 6).

From a histopathological perspective, verruca vulgaris, condyloma acuminatum, and the non-HPV-related lingual mucosal horn each exhibit distinctive epithelial features that aid in differential diagnosis. Verruca vulgaris is characterized by marked orthokeratotic hyperkeratosis, prominent hypergranulosis, and sharply elongated rete ridges converging toward the center. Koilocytosis, when present, is typically sparse and localized. In contrast, condyloma acuminatum demonstrates irregular parakeratosis with minimal granular layer development, broader and more bulbous epithelial projections, and abundant koilocytosis in the spinous layer. Proliferative activity is often reflected by stratified Ki-67 expression beyond the basal layer, although nuclear atypia is generally absent. The lingual mucosal horn, a non-viral reactive lesion, shows compact orthokeratotic hyperkeratosis, centripetal exophytic epithelial hyperplasia with regular rete ridges, and absence of koilocytosis. Immunohistochemically, both p16 and Ki-67 are negative or restricted to the basal layer, further supporting a non-HPV etiology. These contrasting histological profiles are essential for distinguishing between viral and non-viral papillary lesions of the oral mucosa, particularly in cases with solitary, asymptomatic lesions located on the dorsal tongue.

A follow-up evaluation at three months post-excision revealed no evidence of recurrence or mucosal alteration.

## Discussion

Condyloma acuminatum is a benign epithelial proliferation associated with infection by low-risk genotypes of HPV, most commonly types 6 and 11 [4]. Although it is frequently encountered in anogenital regions, its occurrence in the oral cavity is relatively uncommon [5]. Notably, although condyloma acuminatum is primarily transmitted through sexual contact, including oral sexual practices, its manifestation in the oral cavity has historically been considered rare. As early as 1983, Malik et al. reported that only seven histologically confirmed oral cases had been described in the English literature up to that time [6]. A broader review by Zunt et al. in 1989 identified approximately 156 cases in total, highlighting the uncommon nature of this lesion in the oral setting, despite increasing recognition [7]. More recently, Sen et al. reaffirmed the uncommon occurrence of oral condylomas, particularly highlighting their rarity in the elderly population [8]. Within the oral cavity, these lesions typically affect the labial mucosa, lips, floor of the mouth, lateral and ventral surfaces of the tongue, lingual frenulum, buccal mucosa, soft palate, and rarely the gingiva. Involvement of the dorsal tongue is exceedingly rare, likely due to the keratinized nature of the epithelium and the presence of local immune defenses that limit viral persistence. Classically, oral condylomas present as multiple, sessile, exophytic lesions with a papillomatous surface resembling cauliflower or cockscomb morphology [9]. These macroscopic features correspond histologically to broad, blunted rete ridges and prominent koilocytosis in the spinous layer [10]. In the present case, the lesion exhibited several microscopic hallmarks, namely papillomatosis, acanthosis, and koilocytotic changes, despite lacking the typical gross architecture of a florid or lobulated condyloma. Moreover, since binucleation, along with koilocytosis, variations in nuclear size, meganuclei, and abnormal chromatin distribution, have been reported as a reliable morphological marker of HPV-related lesions [11], the cytologic features observed in this lesion further support the diagnosis of condyloma acuminatum. Clinically, the lesion was a solitary, asymptomatic, well-circumscribed papillary projection on the dorsal tongue, measuring less than 2 mm in height and under 1 mm in diameter. Its smooth surface and sharply delimited margins deviated from the expected presentation of condyloma, prompting a broader differential diagnosis that included oral squamous papilloma, verruca vulgaris, and a reactive lingual mucosal horn. Of particular note is the negative result obtained from HPV DNA testing via real-time PCR, which included a comprehensive panel of both high- and low-risk genotypes. This molecular finding introduces diagnostic uncertainty, as it fails to corroborate the histopathological impression. Several possible explanations may account for this discordance, including a false-negative PCR due to viral load under the detection threshold, sampling limitations, or DNA degradation [12-13]. Alternatively, the lesion may reflect a regressed HPV infection with residual epithelial changes or represent a non-viral mimicker of condyloma. Moreover, the HPV family is extremely heterogeneous: not all genotypes are currently covered, and as a result, diagnostic tests are limited to a small portion of them. The differential diagnosis also included verruca vulgaris. Verrucae tend to occur on the lips or anterior tongue and exhibit a keratotic surface with a granular layer and elongated, convergent rete ridges [14]. Our lesion, however, lacked the pronounced hypergranulosis and “church spire” rete ridge architecture characteristic of verruca vulgaris. Reactive epithelial proliferations such as the lingual mucosal horn were also considered, particularly in light of the lesion’s solitary, sharply protruding morphology and lack of viral DNA. These lesions typically show compact orthokeratosis, absence of koilocytosis, and minimal mitotic activity beyond the basal layer, features not entirely consistent with the findings in this case. Although IHC staining for proliferation (Ki-67) and surrogate HPV markers (p16) was not performed, such analyses could have offered additional insight. In condylomas, Ki-67 is often expressed in multiple suprabasal layers, whereas p16 staining tends to be absent or focal due to the low-risk nature of the associated HPV subtypes. The absence of IHC staining in this case, although a limitation, offers an opportunity to assess the diagnostic reliability of classic histomorphological criteria in the absence of molecular confirmation. Taken together, the histological features remain consistent with condyloma acuminatum, despite the negative molecular result and atypical clinical presentation. This case highlights the diagnostic complexities of solitary papillary oral lesions and reinforces the importance of correlating clinical, histological, and virological data in establishing an accurate diagnosis. This case report not only highlights the importance of a multidisciplinary approach, but also reinforces the idea that screening should not be limited to the cervix, but should also be extended to other mucosal sites, such as the oropharynx and the anus, where the virus is increasingly being detected.

## Conclusions

This case illustrates the diagnostic challenges posed by solitary, small papillary lesions of the dorsal tongue, particularly when clinical and molecular findings diverge. Although the lesion lacked the classic exophytic and lobulated morphology, typically associated with oral condylomas, its histopathological features, including papillomatosis, acanthosis, variations in nuclear size, meganuclei, chromatin distribution, binucleation, and koilocytosis, were strongly suggestive of condyloma acuminatum. The negative HPV DNA PCR result, despite testing for a comprehensive panel of high- and low-risk genotypes, underscores the potential for false-negative molecular findings. This raises questions about the interpretation of clearance, which may reflect true viral elimination, a viral load below the assay’s detection threshold, true latency, or inadequate sampling that fails to reach basal epithelial cells. The unusual location and solitary nature of the lesion further emphasize the importance of thorough histopathological assessment, even in the absence of overt clinical suspicion. This case further illustrates the value of integrating multiple clinical and pathological perspectives in diagnosis, while also drawing attention to the growing relevance of HPV detection at non-cervical mucosal sites, including the oropharynx and anus, where routine screening may be warranted. In similar cases, a combined histopathological and IHC approach, including p16 and Ki-67 staining, may enhance diagnostic accuracy.

## References

[1] McCord C, Xu J, Xu W (2014). Association of human papilloma virus with atypical and malignant oral papillary lesions. Oral Surg Oral Med Oral Pathol Oral Radiol.

[2] Gaultier F, Ejeil A-L, Lepelletier F, Gogly B, Cherifi H, Bayet K, Dridi SM (2019). Clinical and histopathological features of a lingual mucosal horn: first time described clinical case series. Dent Oral Craniofac Res.

[3] Betz SJ (2019). HPV-related papillary lesions of the oral mucosa: a review. Head Neck Pathol.

[4] Temple RW, Larker JC, Brus JM (2025). Genital warts: rapid evidence review. Am Fam Physician.

[5] Suskind DL, Mirza N, Rosin D, Stanton D, Sachdeva R (1996). Condyloma acuminatum presenting as a base-of-tongue mass. Otolaryngol Head Neck Surg.

[6] Malik PA, Cabbabe EB, Shively RE, Sunwoo YC (1983). Condyloma acuminatum of the tongue. Ann Plast Surg.

[7] Zunt SL, Tomich CE (1989). Oral condyloma acuminatum. J Dermatol Surg Oncol.

[8] Sen R, Shah N, Sheikh MA, Chatterjee RP (2018). Oral condyloma acuminatum in a 75-year-old geriatric patient. BMJ Case Rep.

[9] Lehnen H (1988). Condylomata acuminata and mode of delivery. Z Geburtshilfe Perinatol.

[10] Diţescu D, Istrate-Ofiţeru AM, Roşu GC (2021). Clinical and pathological aspects of condyloma acuminatum - review of literature and case presentation. Rom J Morphol Embryol.

[11] Kiviat NB, Koutsky LA, Critchlow CW, Lorincz AT, Cullen AP, Brockway J, Holmes KK (1992). Prevalence and cytologic manifestations of human papilloma virus (HPV) types 6, 11, 16, 18, 31, 33, 35, 42, 43, 44, 45, 51, 52, and 56 among 500 consecutive women. Int J Gynecol Pathol.

[12] Groves IJ, Coleman N (2015). Pathogenesis of human papillomavirus-associated mucosal disease. J Pathol.

[13] Syrjänen S (2003). Human papillomavirus infections and oral tumors. Med Microbiol Immunol.

[14] Ural A, Arslan S, Ersoz Ş, Değer B (2014). Verruca vulgaris of the tongue: a case report with a literature review. Bosn J Basic Med Sci.

